# Global lipidomics identified plasma lipids as novel biomarkers for early detection of lung cancer

**DOI:** 10.18632/oncotarget.22391

**Published:** 2017-11-11

**Authors:** Zongtao Yu, Hankui Chen, Junmei Ai, Yong Zhu, Yan Li, Jeffrey A. Borgia, Jin-Song Yang, Jicai Zhang, Bin Jiang, Wei Gu, Youping Deng

**Affiliations:** ^1^ Department of Laboratory Medicine, Shiyan Taihe Hospital, College of Biomedical Engineering, Hubei University of Medicine, Shiyan 442000, Hubei, China; ^2^ Department of Internal Medicine, Rush University Medical Center, Chicago, IL 60612, USA; ^3^ National Center of Colorectal Disease, Nanjing Municipal Hospital of Chinese Medicine, The Third Affiliated Hospital, Nanjing University of Chinese Medicine, Guangdong, Nanjing 210001, China; ^4^ Department of Pathology, Rush University Medical Center, Chicago, IL 60612, USA; ^5^ Department of Oncology, Nanjing First Hospital, Nanjing Medical University, Nanjing, Jiangsu 210006, China; ^6^ Department of Respiration, Nanjing First Hospital, Nanjing Medical University, Nanjing, Jiangsu 210006, China; ^7^ Department of Complementary & Integrative Medicine, University of Hawaii John A. Burns School of Medicine, Honolulu, HI 96813, USA

**Keywords:** lipidomics, lung cancer, early biomarkers, ESI-MS

## Abstract

**Purpose:**

Lipids play roles in membrane structure, energy storage, and signal transduction as well as in human cancers. Here we adopt lipidomics to identify plasma lipid markers for early screening and detection of lung cancer.

**Experimental Design:**

Using mass spectrometry, we profiled 390 individual lipids using training and validation strategy in a total of 346 plasma samples from 199 early NSCLC patients, including 113 adenocacinoma and 86 squamous cell cancers (SqCC), and from 147 healthy controls.

**Results:**

In the training stage, we found distinct lipid groups that were significantly distributed between NSCLC cases and healthy controls. We further defined a panel of four lipid markers (LPE(18:1), ePE(40:4), C(18:2)CE and SM(22:0)) for prediction of early cancer with a accuracy of 82.3% AUC (Area under ROC curve), sensitivity of 81.9% and specificity of 70.7% at the training stage and yielded the predictive power with accuracy (AUC,80.8%), sensitivity 78.7%, specificity 69.4% and in the validation stage.

**Conclusions:**

Using lipidomics we identified several lipid markers capable of discerning early stage lung carcinoma from healthy individuals, which might be further developed as a quick, safe blood test for early diagnosis of this disease.

## INTRODUCTION

Lung cancer is the leading cause of cancer-related deaths worldwide, and accounts for 13% of new cancer cases and 29% of all cancer deaths in the United States each year [1]. Lung cancer is a heterogeneous disease with multiple histological and molecular subtypes, and usually classified according to the histological types that correlate with tumor behavior and prognosis [2]. The vast majority of lung cancer types are non-small cell lung cancers (NSCLC), carcinoma malignancies that arise from epithelial cells and take account for 80% of lung cancers. Of these NSCLCs, there are two most common subtypes: adenocarcinoma (about 70%) and squamous cell lung cancer (SqCC, about 30%). SqCC originates in the large airways in the central part of the lungs, and is the most common histological subtype of lung cancer amongst smokers in European descendants [3, 4]. Detecting lung cancer at earlier stages could reduce mortality rates by 10- to 50-fold [5]. However, this disease is often diagnosed at an advanced stage, and about two-third of patients at the time of diagnosis have metastatic tumors. The current low-dose computed tomography (LDCT) scan approach provides a non-invasive method to detect tumors at early stages, while yields conflicting results [6, 7]. Therefore, it is necessary to develop new minimally invasive methods such as molecular biomarkers for early detection of lung cancer.

Lipids comprise diverse classes of molecules and have numerous critical biological functions in cellular energy storage, membrane structure, and signaling. Lipid levels are tightly regulated, spatially and temporally, in the various parts of the human body. Dysregulation of lipid metabolism contributes to the onset of pathology and progression in a wide variety of human diseases, such as diabetes [8], Alzheimer's disease [9], hypertension [10], and human cancers [11–15]. Aberrant lipid metabolism in lung cancer has also been demonstrated in previous studies, by lipid profiling in twenty-one pairs of resected frozen NSCLCs and adjacent normal tissue samples [15].

The involvement of lipid abnormalities in human diseases raises the potential that lipids could serve as biomarkers for various human diseases. However, due to technical limitations in lipid measurement, only a limited number of studies have studied lipids in this context thus far. Lipidomics is a relatively new field that quantitatively evaluates a range (hundreds) of fat (lipids) species at once, and can be used to produce a lipid profile for most pathophysiological states. Lipidomics has been recently applied as a useful tool in the study of lipid mechanisms in many diseases such as diabetes [16], obesity [17], and some types of human cancers including colon cancer [18], thyroid papillary cancer [19], and prostate cancer [20]. Direct tissue matrix-assisted laser desorption/ionization (MALDI) mass spectrometry (MS) analysis has been used for lipid profiling in resected frozen lung cancer tissue samples [15], however, large scale investigation on plasma lipid profiling has not been reported in lung cancer.

In this present study, we performed a lipid profiling study using tandem mass spectrometry which measured 390 distinct lipids in plasma specimens from early NSCLC patients and healthy controls. The objective of our study is to develop a plasma lipid marker panel for the early detection of lung cancer.

## RESULTS

### Lipid profiling of 390 lipid species in the training cohorts

At the training stage, we identified plasma lipid profiles with measurement of 390 individual apparent lipid species, as defined and annotated on the basis of intact ion-fragment pairs, from 13 classes of phospholipids and cholesteryl esters (CE) by using lipidomics in 185 plasma samples including 105 NSCLC patients and 80 age-, sex-, and race-matched healthy controls. As summarized in Table 2, we detected a total of 361 apparent lipid species of all 13 classes in all training samples. In this study, 29 of 390 lipid species could not be detected in any sample from the training cohorts, mostly from the LysoPE class (14 species) and the PI class (9 species).

**Table 1:** Patients’ characteristics of all samples used in both training and validation stage

	Training		Validation	
	Cancer (105)	Healthy (80)	Cancer (94)	Healthy (67)
**Sex**				
Male	52	40	56	52
Female	53	40	38	15
**Race**				
Caucacian	90	71	79	56
Noncaucacian	15	9	15	11
**Age, yr**				
Median	67	63	67	59.75
SD	8.26	10.24	10.12	11.94
Range	48 – 82	20 – 80	43 – 88	28 – 84
**Smoking history, pack-years**				
Median	30	50	40	20
SD	29.1	26.8	28.8	49.2
Range	3 – 120	5 – 100	1 – 120	17.5 – 104
**Tumor subtype**				
Adenocarcinoma	67		58	
Squamous	38		36	
**Tumor stage**				
Stage I	65		49	
Stage II	40		45	

**Table 2:** The list of lipid classes and lipid species detected at the training stage

Lipid class	Description	No. of lipid species
PC	Phosphatidylcholine	54
PE	Phosphatidylethanolamin	53
PS	Phosphatidylserine	51
PI	Phosphatidylinositol	46
ePC	PC with one ether-linked (alkyl or alkenyl) chain	27
ePE	PE with one ether-linked (alkyl or alkenyl) chain	27
PA	Phosphatidic acid	22
ePS	PS with one ether-linked (alkyl or alkenyl) chain	19
CE	Cholesterol esters	18
LysoPC	Lyso-Phosphatidylcholine	14
LysoPE	Lyso-phosphatidylethanolamin	13
SM	Sphingomyelin	12
PE-cer	Ceramide phosphoethanolamine	5

A total of 13 lipid classes and 361 lipid species were detected in the training samples.

### Identification of lipid species significantly differentiated between NSCLC patients and healthy individuals

In order to select individual apparent lipid biomarkers from hundreds of detected species, we first use a filtration strategy to narrow down the number of potential candidates from 361 apparent lipid species. In this step, we excluded those lipid species that cannot be clinically used in diagnosis of NSCLC disease due to too low concentration to detect, or insignificant difference between patient and control groups, or too closed levels of plasma concentrations in two groups to interpret (although the difference may be statistically significant). Criteria for retention were: 1) difference in mean plasma lipid concentration is significant (*p* ≤ 0.05) between patient and control groups; and 2) changes in mean plasma lipid concentration is ≥ 10% (up or down); and 3) mean plasma lipid concentration is ≥ 10 nmol/μL. Using this strategy, we obtained a list of apparent lipid species that fulfilled all the three criteria in the training samples, which could be selected as potential candidates of plasma lipid biomarkers for NSLCL.

### Identification of a panel of lipids as candidate biomarkers for early-staged NSCLC

We then use the second strategy provided additional differentiation of cancer and control samples, in order to demonstrate that the selected candidates are not only clinically useful and applicable, but also they are highly sensitive, specific and accurate in differentiation of NSCLS from healthy controls. After analysis with bioinformatics methods, any apparent of lipid species of selected potential candidates will be selected as individual plasma lipid biomarker in diagnosis of NSCLC cancer, if it met these criteria: 1) sensitivity above 80%; 2) specificity above 50%; and 3) area under (ROC) curve above 80%. However, we have not identified any single lipid species meeting these criteria even though it is significantly different between cancer patients and normal controls.

**Figure 1:**
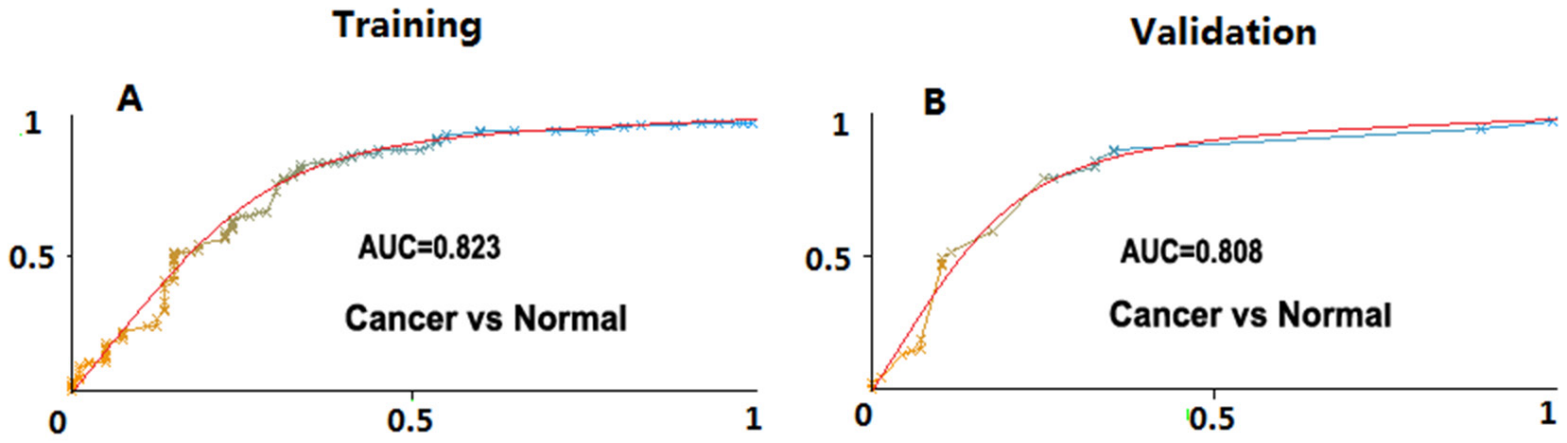
Area under the curve (AUC) values of lipid panels for disease prediction in training and the validation cohorts The AUC values in disease prediction were shown for the lipid panel in distinguishing NSCLC from healthy controls at the training and the validation stages, respectively.

We then used combination strategies to search for the combined lipid molecules who can meet the criteria described above. We finally determined a panel of four lipid species, including LPE (18:1), ePE(40:4), C(18:2)CE and SM(22:0), as candidate biomarkers for early detection of NSCLC disease at the training stage (Table 3). LPE (18:1) and ePE(40:4) showed significant increase of concentration in NSCLC cases as compared to normal controls, while C(18:2)CE and SM(22:0) showed decreases (Table 3). The predictive power of this lipid panel in diagnosis of early stage NSCLC was shown with Area Under Curve (AUC) of 82.3%, sensitivity of 81.9% and specificity of 70.7% at the training stage (Table 4 and Figure 1A) that 105 cases and 80 normal individuals were analyzed.

**Table 3:** Top significant lipid species and marker panels for lung cancer prediction

Lipid type	Training stage			Validation stage		
	P-value	FDR P	FC	P-value	FDR P	FC
LPE(18:1)	5.00E-04	1.70E-03	1.88	1.02E-06	2.03E-04	2.05
C(18:2)CE	3.32E-03	2.69E-02	-1.19	1.38E-01	2.34E-01	-1.18
ePE(40:4)	4.35E-03	3.51E-02	1.27	7.14E-02	1.27E-01	1.24
SM(22:0)	8.40E-03	4.36E-02	-1.30	4.27E-02	9.78E-02	-1.23

FDR P, P value after False Discovery Rate adjusted; FC, Fold change; Minor FC stands for decreasing.

**Table 4:** Predictive values of lipid panels at the training and the validation stage

Stage	Sample size	Sens	Spec	PPV(%)	NPV(%)	AUC	OR(95% CI)	Power
Training	105 vs. 80	0.819	0.707	79.63	73.61	0.823	10.9(5.4 −22.0)	0.982
Validation	94 vs. 67	0.787	0.694	77.08	71.43	0.808	8.4(4.2-17.0)	0.971

The predictive values of four lipid species were analyzed between NSCLC vs control. Sens., sensitivity; Spec., specificity; PPV, positive predictive value; NPV, negative predictive value; OR, odds ratio; AUC, area under the curve.

We further tested the lipid marker panel in our independent validation cohorts including 94 cases and 67 healthy controls. Our data showed the similar pattern of the concentration change of the four lipids in these validation samples as compared with training samples (Table 3). At the validation stage, we also observed the combination of these four lipid markers yielded the predictive power with sensitivity 78.7%, specificity 69.4% and accuracy (AUC,80.8%), as shown in Table 4 and Figure 1B.

## DISCUSSION

In this present study we conducted an extensive plasma lipidomics profiling in NSCLC patients and identified a distinct panel of lipid biomarkers which can predict the NSCLCS at early stage. In the targeted mass spectrometry approach that was utilized, the predicted biomarkers have an intact ion and fragment consistent with the indicated identifications; it should be noted, however, that these identifications are preliminary. To date, this is the first original report on plasma lipid biomarker for the purpose of early detection in lung cancer. Plasma is ideal to develop a quick, non-invasive blood test for early diagnosis of this disease, and our results showed the potential of the four lipid markers used as a companion test of LDCT-based screening methods to distinguish NSCLC patients from high-risk individuals.

**Figure 2:**
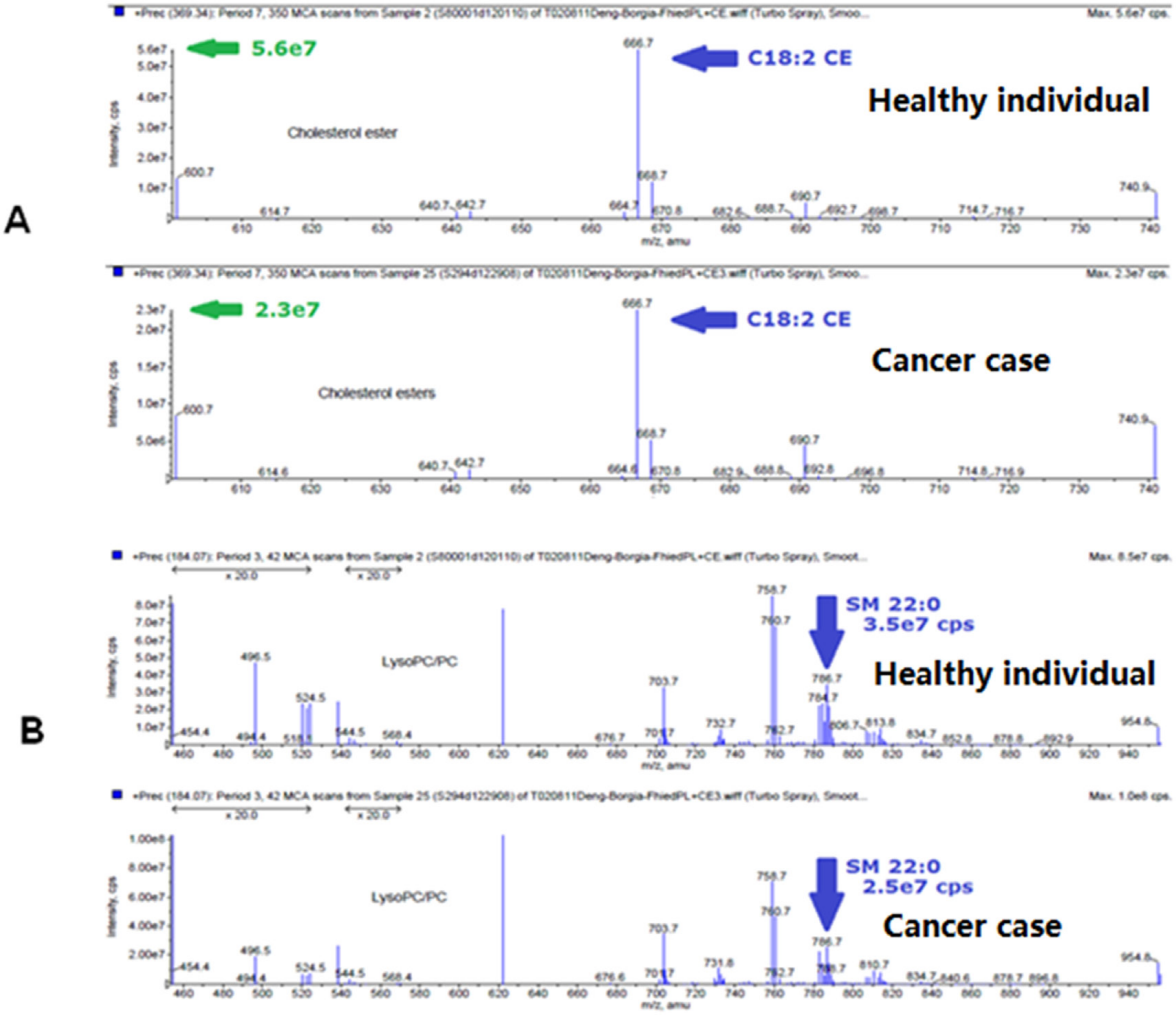
Mass-spectra examples of C18:2 CE and SM 22:0 level in SqCC patients and high-risk controls Spectra of C18:2 CE **(A)** and SM 22:0 **(B)** in a representative healthy individual and a representative cancer patient.

Lipids play an important role in biological functions, including membrane composition and regulation, energy metabolism, signal transduction, etc. It is not surprisingly that they have been found to be involved in cancer [11–15]. In this study, we also observed that NSCLC patients had decreased plasma lipid concentrations of the two lipid molecules, C(18:2)CE and SM(22:0), when compared with healthy controls (Figure 2). The cholesteryl linoleate C(18:2)CE is identified as one of the three major cholesteryl esters present in human low-density lipoprotein (LDL), and the oxidization of C(18:2)CE is believed to be correlated with atherosclerosis [16]. Recently it has been shown that cholesteryl linoleate C(18:2)CE can be oxidized to form compounds 9-ON-secoA and 9-ON-secoB, both of which have been found to exhibit strong cytotoxicity against human leukemia HL-60 cells [17]. However, it is still unclear if the oxidization of C(18:2)CE is involved in tumorogenesis of lung cancer. Even though smoking history is believed to be a risk factor of NSCLC, no association of plasma concentration in cholesteryl esters has been observed between smokers and non-smokers at a large scale of lipidomics analysis [24]. In our study we have not seen the correlation between the smoking history and the lipid level of C(18:2)CE in all plasma samples.

In humans, sphingomyelin (SM), also called as sphingophospholipid, is a type of sphingolipid found in cell membranes and represents ~85% of all sphingolipids. Sphingomyelin consists of a phosphorylcholine head group, a sphingosine and a fatty acid tail. The sphingosine and fatty acid can collectively be categorized as a ceramide. This composition allows sphingomyelin to play significant roles in signaling pathways [25], and the degradation of sphingomyelin can produce ceramide which is involved in the apoptotic signalling pathway [26]. In fact, ceramide can be readily converted to sphingosine 1-phosphate (S1P) or to ceramide 1-phosphate (C1P), whereas both S1P and C1P have opposing effects to ceramide in the regulation of cell growth and survival, acting as pro-survival or mitogenic signals in most cell types and in controlling tumor progression and metastasis as well [27]. Ceramide is a well-studied sphingolipid in both normal and pathological conditions ranging from skin development to lung cancer. In a recent nested case-control study, higher concentrations of S1P and total ceramide in plasma were observed to be associated with increased risk of lung cancer [28]. Several groups have also previously reported increased ceramide levels in high risk smokers, whereas the molecular mechanisms through which cigarette smoke and ceramide accumulation lead to lung cancer, are still largely unknown [27]. Recently two signaling pathways through (1) neutral sphingomyelinase2 (nSMase2, an enzyme that hydrolyzes sphingomyelin to ceramide) or (2) EGF receptor (EGFR), which may actually converge and integrate, have been demonstrated for the biological process during the exposure of cigarette smoke in the lung airway, with the observation that EGFR is favorably co-localized in ceramide-enriched regions of the plasma membrane [29, 30].

In our study, LPE(18:1) was shown significantly related to disease status with increasing level in NSCLC patients, mainly in adenocarcinoma cases. Lysophosphatidylethanolamine (LPE) is a group of signalling lipids, and it has been recently shown to be related to breast cancer [31]. In addition, we observed egg phoshphatidylethanolamine (ePE) as a major lipid group present in plasma, and ePE(40:4) varied between cancer patients and healthy controls in this study. While, the conclusion of these lipids as potential biomarkers for NSCLC prediction still needs to be confirmed in more samples from different resources.

In summary, we report that four lipid species could distinguish early-staged NSCLC from healthy individuals based on our observation using the training and the independent validation cohorts. Limitations of this study are that we have not included the benign patient samples and that we did not know if these lipids are specific to the status of lung cancer disease. With the information gained from our study, we will continue using the lipidomics strategy in a larger data-set of normal, benign and NSCLC patient plasma samples to validate our findings.

## PATIENTS AND METHODS

### Patient cohorts

*Training cohorts*. We enrolled approximately 1,250 patients in our Lung Cancer Biorepository at Rush University Medical Center (Chicago, IL) between 2004 and 2010 and of these selected a sub-cohort of 105 patients with early staged (stage I, II) NSCLC, including 60 adenocarcinoma and 45 SqCC case, and 80 healthy individuals for this pilot study. The early stage NSCLC patient inclusion criteria included the disease confined to the chest without evidence of distant metastases; no preoperative chemo- or radiotherapy within 1 year of our initial blood sampling; and a minimum of 2 years of clinical follow-up data. Healthy individuals were aged 55 to 75 years, followed with annual LDCT and remained cancer-free for a minimum 2-year follow-up. Demographic information for these patients and controls is listed in Table 1. All patient data were acquired with written formal consent and in absolute compliance with the institutional review board at Rush University Medical Center.

*Validation cohorts:* We used independent cohorts of 161 plasma samples, including 94 patients with early-staged NSCLC (53 adenocarcinomas and 41 SqCCs) and 67 healthy individuals in the validation stage. These cohorts were obtained from Lung Cancer Biospecimen Resource Network (LCBRN) at University of Virginia, and the inclusion criteria of cases and controls was the same as used in training cohorts. All plasma samples were collected using EDTA-anticoagulative tubes and centrifuged for at 4000 RPM for 10 min, followed by a 15 min high-speed centrifugation at 12,000 RPM to completely remove cell debris. The supernatant plasma was stored at −80°C until analysis.

### ESI-MS lipid profiling

Electrospray ionization-mass spectrometry (ESI-MS) as a sensitive and powerful technology in lipidomic applications for disease biomarker discovery [20, 21, 22], in this study we used ESI-MS system to detect a total of 390 lipids in plasma samples at Kansas Lipidomics Research Center (Kansas State University, Manhattan, KS), as described previously [20, 23]. Briefly, an aliquot of 3 μL of plasma was used, and plasma lipid species were identified at level of head group plus total acyl carbons: total double bonds in this assay, with precise amounts of internal standards obtained and quantified, as previously described [20]. Sequential precursor and neutral loss scans of the extracts produced a series of spectra with each spectrum revealing a set of lipid species containing a common head group fragment. The samples were continuously infused and that the internal standards were measured under the same conditions as the biological lipids. A total of 13 lipid classes containing 390 species were measured: PC, SM, lysoPC, PE, lysoPE, PI, PS, PA, CE, SM, ePCs, ePEs and PE-cer, as detailed previously [20]. The background of each spectrum was subtracted, the data were smoothed, and peak areas integrated using a custom script and Applied Biosystems Analyst software. Finally, the data were corrected for the components of the sample analyzed and normalized to the sample volume to produce data in the unit of nmol/μL.

### Statistical and bioinformatics analysis

In this study we used the T-Test in SPSS 18 software to compare mean plasma concentrations of 390 lipid species between all NSCLC cases, adenocarcinoma, SqCC patient and control groups after the data were log transformed, with the significant p value set at 0.05. Simple logistics classification algorithm and InfoGain were used to rank individual apparent lipid species and lipid class according to their predictive powers in NSCLC patients, and 10-fold cross validation were used to estimate the performance of a predictive model. Chi-Square test in SPSS 18 software was used to compare the distribution of controls and patients with regards to plasma lipid concentrations, and the significant p value was set at 0.05 for all results from Chi-Square tests.

### Translational relevance

Cancer screening allows the detection of early-stage tumors and is helpful to reduce mortality of this disease. Plasma lipids represent a class of molecules being utilized as potential blood-based marker for human cancer screening. In the current study, we conducted a global lipidomics assay anddemonstrated for the first time, in a training cohort and an independent validation cohort of patients with non small cell lung cancer (NSCLC), one panel of lipid markers, can be used as noninvasive biomarker for the diagnosis of NSCLC. Our results could be developed as a screening approach for the early detection of lung cancer.
